# Is the expression of different discrete emotions related to time? Evidence from online Chinese reviews using sentiment analysis and human behavior dynamics

**DOI:** 10.3389/fpsyg.2024.1321582

**Published:** 2024-03-06

**Authors:** Liu Li-na, Qi Jia-yin, Wang Sheng-feng, Zhang Zhen-ping, Qu Qi-xing

**Affiliations:** ^1^School of Modern Post (School of Automation), Beijing University of Posts and Telecommunication, Beijing, China; ^2^School of Cyberspace Security, Guangzhou University, Guangzhou, China; ^3^School of Information and Communication Engineering, Beijing University of Posts and Telecommunication, Beijing, China; ^4^International School, Huaqiao University, Quanzhou, China; ^5^School of Information, University of International Business and Economics, Beijing, China

**Keywords:** discrete emotions, sentiment analysis, human behavior dynamics, “purchase-comment” time intervals, online Chinese reviews

## Abstract

**Objectives:**

The online behavior of online users has taken on complex and diverse characteristics, and posting product reviews on e-commerce platforms is no exception. In fact, reviews contain rich and multi-dimensional discrete emotional information, and whether there is a relationship between the expression of these different discrete emotions and the time interval between product purchase and review posting as well as their related characteristics are the issues that this study needs to analyze and solve in depth.

**Methods:**

Based on the OCC model (named after three proposers) of psychological emotional cognitive evaluation theory as the basis for emotion classification, the study used the massive amounts of Chinese reviews of mobile phones on the Chinese e-commerce platform Jingdong Mall as the research object, applied supervised machine learning methods to classify discrete emotions, and constructed a large corpus containing satisfaction, disappointment, admiration, reproach, love, and hate; then the study delved into the distribution and behavioral dynamics characteristics of consumers’ comments containing the different discrete emotions at different “purchase-comment” time intervals.

**Results:**

The results showed that the first peak of the distribution curves of the six discrete emotions at different “purchase-comment” time intervals occurs on the first day after purchase and then decreases gradually but at different rates. The three curves for satisfaction, love, and hate emotions also show a second peak on the eleventh day, which is more similar to the bimodal distribution, implying that the corresponding product reviews are more objective. In addition, the distribution of reviews containing the six discrete emotions at different “purchase-comment” time intervals follows a power-law distribution and has the temporal characteristics of human behavioral dynamics, that is, “strong paroxysms and weak memory“. However, the reviews containing the admiration and reproach emotions were most intensively written by consumers after the purchase, indicating that the service provided by the seller, logistics, and e-commerce platform stimulates more consumers to give quick responses and detailed reviews.

**Conclusion:**

This study is not only of great significance for exploring the internal mechanisms of consumer discrete emotional expression but also provides important decision-making references for potential consumer purchasing decisions, product updates for developers, marketing strategy formulation for marketing teams, and service improvement for sellers, logistics companies, and e-commerce platforms.

## 1 Introduction

With the coming of the Web 2.0 era, user centered methods are gradually replacing traditional methods, spreading data through user interest in content and value creation. The network behaviors of online users present complicated and different qualities. Among all human online activities, posting online product reviews is an important part of e-commerce. Online product reviews have become an important medium for communicating and disseminating product content, giving feedback to users’ questions and opinions about products, and promoting the continuous improvement of products and services by merchants. With the growing increase of online product reviews and increasingly complicated content forms, manually reading, analyzing, and processing information is time-consuming and arduous. In this context, the study of sentiment analysis of online reviews has emerged. Existing online reviews of sentiment analysis studies generally classify emotions into positive and negative emotional polarity (Power and Dalgleish, 1999; Xu, 2013) or into positive, negative, and neutral emotions (Zhao et al., 2010). With the deepening of research, more and more scholars have found that valence analysis cannot characterize human emotion well nor can it help us to deeply understand natural language. Valence analysis ignores the richness and multi-dimensional structure of human emotion (Cambria et al., 2013). In fact, discrete emotion theory, which originated in the 1870s, states that some core emotions are biologically determined emotional responses that remain consistent across ethnic and cultural backgrounds. Ekman and Izard argued that through verbal and expressive transmission, humans express a basic set of emotion categories, including anger, disgust, fear, joy, sadness, and surprise (Ortony et al., 1988; Power and Dalgleish, 1999); other emotions can be made out of the six basic emotions, which are considered the universal cognitive outcome of human emotions. Indeed, Internet comments provide a wealth of rich, multidimensional sentiment data (Xu, 2013). The emotion classification algorithm based on discrete emotion theory is more comprehensive and specialized than the other major methods described above. In this context, in recent years, some scholars have begun to pay attention to the economic value of discrete emotional information in online comments. Most of the relevant literature adopts survey questionnaires or experiments to investigate several perspectives such as the perceived usefulness of different discrete emotions on reviews (Yin et al., 2014; Ahmad and Laroche, 2015; Gang and Taeho, 2018; Craciun et al., 2020), the spread of discrete emotions in online reviews (Xiao et al., 2017; Mokryn et al., 2020), and the impact of discrete emotions in online reviews on product sales (Yu et al., 2023). Although there are not many existing studies, most of the results show that different discrete emotions, even if they have the same valence, exhibit different economic values. It can be seen that analyzing discrete emotions in online reviews can help us better understand the psychology of consumers in e-commerce and have a more in-depth look at the emotions of consumers and their influencing factors. This is critical for establishing consumer-centric marketing strategies, increasing the competitiveness of items on the market and improving the services of goods sellers and e-commerce platforms.

Human behavior has long drawn in the consideration of researchers in many fields, including sociology, psychology, and anthropology. However, due to the limited data collection and processing ability of human activities, most of these early studies were qualitative studies, and the establishment of human behavior theory from a quantitative perspective has become the main interest of contemporary science (Li et al., 2008). In 2005, Barabási published an article in Nature which showed that the temporal patterns of human behavior are highly non-uniform, with very long periods of time in which nothing happens, while these long gaps are filled with bursts of intensive activities (Barabási, 2005). Barabási’s work opened up a new field of research called the dynamics of human behavior. Although this field has not been around for a long time, many scholars in the fields of mathematics, systems science, and statistical mechanics have published a great deal of research results showing its theoretical and practical value. They used data mining algorithms and data analysis technology to study individual or group behavior in many fields, such as mail sending and receiving (Oliveira and Barabási, 2005), commercial trade (Vázquez et al., 2006), book borrowing (Fan et al., 2012), and logistics and transportation (Wang and Guo, 2010), and they found that human behavior cannot be characterized by the traditional Poisson process, which means that the time interval of behavior occurrence follows a negative exponential distribution, and the number of events follows the Poisson distribution. Instead, the time interval of behavior occurrence follows the power-law distribution and has the characteristics of bursts and a heavy tailed distribution. In view of the fact that online activities are an important part of human behavior, many scholars have explored the time interval law of human online activities through the idea of behavioral dynamics, including web browsing (Dezsö et al., 2006), movies on demand (Zhou et al., 2008), online games (Henderson and Bhatti, 2001), online reviews (Wang et al., 2010), and blog and microblog publishing (Guo et al., 2011; He et al., 2013). Some scholars believe that the temporal characteristics of online purchase and review behavior can still be analyzed using the theoretical method of behavioral dynamics, and the power index can be calculated to determine whether the online review behavior also conforms to the characteristics of behavioral dynamics. The existing research on the time interval distribution of online users’ “purchase-review” is primarily focused on different types of products (Wang and Guo, 2010; Zhang, 2018), and whether there is a link between the time interval of the “purchase-review” and distinct discrete emotions, that is, whether the expression of different discrete emotions in reviews is influenced by the time of purchase and release of reviews, whether the “purchase-review” time interval of reviews containing different discrete emotions still follows a power-law distribution, and whether there are similarities and differences, are issues to be analyzed and solved in depth in this study. It can be foreseen that analyzing the relationship between the expression of different discrete emotions and the time interval between product purchase and comment posting, exploring their related characteristics, is important for exploring the internal mechanisms of consumer discrete emotional expression and providing important decision-making references for potential consumer purchasing decisions, product updates for developers, marketing strategy formulation for marketing teams, and service improvement for sellers, logistics companies, and e-commerce platforms.

The rest of the paper is organized as follows. In Section 2, we review the research related to discrete emotion theory, textual sentiment analysis, and network behavior based on the dynamics of human behavior. Section 3 presents a discrete emotion classification method for Chinese online reviews. Section 4 examines how people express discrete emotions in online reviews using human dynamics. Finally, the paper concludes and gives the theoretical and practical implications in Section 5.

## 2 Related work

### 2.1 Discrete emotion theory

Discrete emotion, also known as basic emotion, comes from the discrete emotion theory, which believes that all the emotions can be derived from a limited number of universal and innate basic emotions. In fact, Darwin (1872) was the first to point out that “some emotions have distinctive characteristics.” He described the facial characteristics, physiological and behavioral processes related to different emotions of human and animals, and elaborated the criteria used to explain the functions of these processes and their evolutionary reasons. Similarly, Dewey (1894) also believed that emotions are related to different neural and physiological processes, different functions, and different experiences. Furthermore, we can see similar views earlier. For example, in De Anima, a psychological work, Aristotle (1986) observed that a change of the body is always accompanied by a change of emotion. In the Passions of the Soul, Descartes (1989) described in detail the various physical processes accompanied by different emotions of human beings, as well as the various feelings produced by these physical processes in the mind. He also pointed out that emotional physical processes have specific survival related and self-regulation functions, and he even tried to explain why specific physical processes are accompanied by specific experiences. Tomkins (1962, 1963) was influenced by Darwin’s views. He believed that there are a limited number of eight pan-cultural basic emotions (surprise, love, joy, anger, fear, disgust, shame, and pain) and their corresponding “emotional processes.” A few years later, Ekman and Friesen (1971) and Izard (1987) conducted a series of cross-cultural studies to test Tomkins’ hypothesis. They pointed out that regardless of ethnic and cultural differences, people around the world produce and recognize similar facial expressions of six basic emotions (happiness, anger, sadness, disgust, surprise, and fear). Since then, it has been assumed that there are a limited number of pan-cultural basic emotions in emotional psychology, known as discrete emotion theory. The adjective “discrete” means that these basic emotions are independent and unique and can be distinguished from each other according to different characteristics. Although Ekman and Izard only focused on the differences in facial expressions, some other subsequent studies further revealed that there were certain differences in the activity patterns of the autonomic nervous system (Levenson, 2002), neural and chemical processes (Panksepp, 1998), and voice expression (Scherer et al., 2003) corresponding to different discrete emotions.

The “discrete emotion” referred to in this study is based on the “discrete emotion theory” in psychology, which refers to some basic emotions expressed by consumers in online comments on the quality, performance, and user experience of specific goods. These emotions can not only make commentators produce different facial expressions, voice expressions, and different autonomic nervous system activities and behavior patterns but they can also be “diagnosed” and “read out” by other potential consumers to influence their purchasing decision behavior.

### 2.2 Research on text emotion recognition

In recent years, more and more emotional information has appeared in microblogs, blogs, online forums, and online commodity reviews. Many online news sites and social media communities even provide emotional voting services to enable users to express their emotional state after reading news articles (Bao et al., 2009). These data not only effectively convey the positive or negative emotions of Internet users but also express their more specific emotions, such as happiness, fear, or surprise (Shaikh et al., 2008). Emotion recognition technology comes into being, which aims to identify different emotions from subjective texts. This technology is developed from the emotional analysis of human faces and speech and has become the youngest branch in the field of affective computing research. Emotion recognition technology not only attracts the attention of scholars in machine learning and natural language processing but also reflects its importance more and more. It has a very wide range of applications in education, political prediction, commodity marketing, and human–computer interaction.

In fact, emotion recognition technology is challenging because the information embodied in the text itself is very limited and very vague. The text can only capture part of the emotions that human beings can express (Schwarz-Friesel, 2015), and it depends largely on the context and common sense. In different contexts, different purposes and meanings of the same word may express different emotional states. There are a variety of methods used in existing research on emotion recognition, most of which are the same as the subjective text sentiment tendency classification method. They are mainly divided into the following four methods: the keyword-based method, dictionary/corpus-based method, machine learning method, and hybrid-based methods.

The keyword-based method is the simplest method to identify the emotion of text, which uses emotion keywords to determine the emotional state of the text. This method takes the text document of one or more sentences as input, detects and extracts emotional keywords after word segmentation, finds the frequency of emotional words through simple statistics, and outputs a specific emotional class. The dictionary/corpus-based method can be regarded as an extension of the keyword-based method, and its main idea is to assign a weight to all emotion words of a certain emotion category, in which the basic emotion is at the top of the emotion ontology list and has a higher weight while the compound emotion is at the bottom of the ontology list with a lower weight. Emotion classification is carried out by counting the weight of different emotion words in each category of emotion in one or more sentences. The method based on machine learning learns from the data itself and finds the relationship between the given input text and the corresponding output emotion by building a prediction model. This method overcomes the limitations of the keyword-based method and dictionary/corpus-based method and can be divided into a supervised learning method and unsupervised learning method. The supervised learning method is to label or annotate a part of the dataset, train it using emotion classifiers, then analyze the training results and build a model, finally classifying the rest of the dataset according to the trained classifier, so as to get the emotion category. The unsupervised learning method is a self-learning classification method, which aims to discover the hidden structural relationship between unknown data. Although this method saves a lot of time regarding manual annotation, it needs a huge dataset to realize emotion classification. The last method, hybrid-based methods, uses the combination of the above methods to improve the accuracy of text emotion recognition. Table 1 lists some of the literature on text emotion recognition in recent years.

**Table 1:** Selected literature on text sentiment recognition in recent years.

Literature	Language	Recognition technology	Datasets
Shivhare et al. (2015)	English	Keywords and emotional ontology	135 blogs
Wang and Pal (2015)	English	Sentiment dictionary	SemEval, ISEAR, Twitter
Rana and Kolhe (2015)	English	Machine learning-based: SVM and plain Bayesian multilayer label classifier	Twitter
Lee and Wang (2016)	English and Chinese	Machine learning-based: maximum entropy classifier	Data from Weibo.com
Rao et al. (2016)	English	Topic-level maximum entropy model	BBC, Myspace, Twitter, YouTube, etc.
Almeida and Paraiso (2017)	Portuguese	Supervised machine learning methods	Newspaper headlines
Tareaf et al. (2018)	English	Machine learning	Facebook
Chatzakou et al. (2017)	Multi-language	Hybrid learning method	News, Twitter, and Facebook
Xu et al. (2019)	Chinese	Sentiment dictionary	Review Data on Ctrip and Jingdong Websites
Cheng et al. (2020)	English	Machine learning-based: deep learning	IMDB dataset and Yelp 2015 dataset
Han et al. (2020)	English	Machine learning	Twitter
Butt et al. (2021)	English	Machine learning	PHEME dataset
Minzheng et al. (2021)	Chinese	Machine learning-based: neural network	Sina Weibo
Sultana (2022)	Telugu	Machine learning	Telugu News dataset from Kaggle

### 2.3 Research on the studies of network behavior based on the dynamics of human behavior

The huge community of users on the Internet generates huge amounts of data every day, recording the rich and diverse online behavior of a large number of users, and an in-depth exploration of online behavior poses new challenges for current research on human behavior. Scientifically speaking, what the similarities and differences between online behavior and other human behaviors are, as well as the mechanisms behind them, are intriguing but not yet fully explored; while in terms of application, an in-depth study of the universal laws of online behavior can help to understand various social phenomena at a deeper level, which is of great significance for public opinion analysis, network management, e-commerce marketing, and so on.

The current research on human network behavior regards the occurrence of behavior as a random process of “task” arrival and mainly focuses on the statistical law of behavior at the time level, which is usually characterized by the time interval of behavior occurrence. The traditional view holds that the occurrence of behavior is random and uniform, and human behavior is described by the Poisson process. A large number of empirical studies also show that some human online behaviors, such as online social networking (Grabowski et al., 2008; Rybski et al., 2009; Zhao et al., 2013; Mathews et al., 2017), movies and music on demand (Crane and Sornette, 2008; Hu and Han, 2008; Zhou, 2008), and web browsing (Dezsö et al., 2006; Zhao et al., 2011; Zha et al., 2016), always occur intensively in a short time after a long period of silent waiting. The interval time or waiting time of behavior shows a strong fat-tail characteristic of deviations from Poisson processes and can be best described as a power-law distribution. In addition, although Vázquez et al. found that the Barabási model can only produce two discrete power functions with a power index of 1 or 1.5 through their analytical research, more and more empirical studies have proved that this speculation may be incorrect.

In addition to the above human online activities, some scholar also analyzed online purchase or comment behavior using the theoretical method of behavioral dynamics. Wang and Guo (2010) collected the data of four types of products from 360buy.com, including computer products, daily necessities, digital products, and household appliances. They conducted an empirical analysis and found that both the time interval of continuous purchases and the time interval between purchase and comment almost follow the power-law distribution, and the power-law index of the latter is about −1.7. Zhang (2018) selected the relevant information of search and experience goods, including digital products, clothing and shoes, cosmetics, and books, to conduct experimental analysis. It was found that the time interval between the purchase and comment of each type of product conformed to the human behavior dynamics model, the power index was from −1 to −2 , and the time interval also showed a strong paroxysm and weak memory. Wang et al. (2019) used online reviews on movie review websites as a data source to construct a time interval sequence for online reviews. Based on concepts related to human behavioral dynamics, they explored from the perspective of review text length and found that although the time interval sequences of different types of online review behaviors follow a power-law distribution and have certain similarities, they also show significant differences. Zhang et al. (2019) used the initial and follow-up reviews of online comments on JD.com mobile phones as data sources to reveal the power-law distribution characteristics of the lag in the time series of online additional comments from a time dimension. They also explored the content of comment texts in different time series intervals and summarized the time series correlation characteristics and user behavior patterns of online user follow-up behavior.

Due to the complexity and diversity of human behavior, understanding human activities is still a long-term challenge. Although many human behaviors have been studied, many studies have found non-Poisson characteristics of human behavior, so do all human behaviors have such non-Poisson characteristics? As an important part of human online behavior, online shopping has attracted the attention of many scholars. In the existing studies, the analysis of distribution characteristics of time interval between purchase and comment is mostly on the basis of various kinds of products. Whether the expression of different discrete emotions in reviews is affected by the time of purchase and release of reviews, whether the time interval between the purchase and comment of reviews with different discrete emotions still follows the power-law distribution, and whether there are similarities and differences in the above distribution are the problems to be analyzed and solved in depth in the paper (Table 2).

**Table 2:** Some empirical research based on human behavior dynamics in recent years.

Literature	Human behavior	Data sources	Distribution character
Yang et al. (2018)	Repurchase	The public dataset of dianping.com website, which is the leading local life information and trading platform	The time interval distribution of repurchase behavior follows the power-law distribution and the alterable exponent of the power law distribution for different individuals.
Zhang (2018)	Purchase and comment	The online comment dataset of 28 products in JD Mall (www.jd.com), including mobile digital products, clothing, shoes and hats, cosmetics, and books	The time interval between the purchase and comment of each type of products conformed to the human behavior dynamics model, and the power index was from to-1 to-2.
Yu (2020)	Shared bicycles	The experiment dataset from Mobike company records in May 2017 in Beijing area, which contains 3 million travel records data, covering more than 300,000 user IDs and 400,000 bicycle running logs	The shared bicycle’s user behavior obeys the power-law distribution. In the time interval, user behavior with shared bicycles has strong intermittency and weak memory.
Yi et al. (2020)	Verified users’ (VUs) posting on Sina micro-blog	A large-scale behavioral dataset involving 495 VUs and five topics on Sina micro-blog	Three important characteristics of VUs’ micro-blog posting behavior are observed: fat-tailed distribution, fluctuation, and periodicity.
Li et al. (2020)	Posting	A dataset from Sina microblog, containing 46,368 microblog users and more than 0.15 billion tweets.	The follower number distribution of elite users does not fit the power law while that of civilian users does. The inter-tweeting time distributions of different user stratum are similar on a minute scale but have significant difference on a day scale.
Zhang et al. (2020)	Moment posting	A dataset from WeChat, containing 10,196 Moments of 628 users.	Both the number of commits and the inter-event time conform to the power-law distribution in a continuous period of time, and the WeChat users have a low-burstiness, low-memory posting pattern.
Rashidisabet et al. (2022)	Typing	A unique smartphone typing dataset that was passively and unobtrusively collected in-the-wild from 296 users via a custom-made iPhone keyboard.	The power-law is a plausible candidate to represent human typing dynamics.

## 3 Discrete emotion analysis in online reviews

### 3.1 Selection of emotion model

It is critical to use the right discrete emotion model to investigate discrete emotions implicit in online comments. A too complex emotional model causes learning difficulties and low feasibility, otherwise an emotion model that is overly simplistic causes more uncertainty in the emotion category mapping, as well as a bad effect of emotion classification. Many scholars, both domestic and international, have conducted various studies on the categorization of customers’ discrete emotions based on relevant psychological and marketing theories, but there is no uniform standard as of yet. The cognitive evaluation theory in psychology is one of the most well studied ideas in the existing emotional literature, and according to this view, emotion is triggered by people’s subjective assessments of a significant occurrence (Deci and Ryan, 1985).

The OCC model was first proposed in 1988 by Ortony, Clore, and Collins in their co-authored book The Cognitive Structure of Emotion, which is not only one of the most influential models in cognitive evaluation theory but also the first structured model in the field of AI. In order to carry out the computation of the emotion model effectively, the OCC model proposes a scheme for classifying emotions based on cognitive induction conditions and gives the corresponding inferential process. Specifically, this means that people react to the world in three ways, based on whether the outcome of the event is pleasant, whether the agent in the event is satisfying, and whether the object in the event is endearing. The different branches are distinguished due to different intensities or incentives, and 22 different types of emotions are summarized. Given the good computability of the OCC model, the OCC model was chosen as the discrete sentiment model for this study, thus laying the foundation for further research on discrete sentiment in online reviews. In addition, the cognitive induction mechanism of emotion in the OCC model assumes a significant part in supporting the investigation of the causes and behavioral dynamic mechanisms of discrete emotions in online comments.

**Figure 1:**
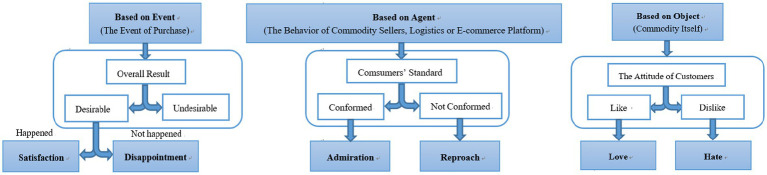
The recognition pattern diagrams of the six-dimensional discrete emotion model.

Although the OCC model divides people’s emotional states into 22 different categories, in practice, online reviews of mobile phones in e-commerce platforms are unlikely to encompass all emotions. Conati (2002) pointed out that the emotional states of users that should be identified in the computer system are “those emotions that have a significant impact on users’ behaviors in the system.” This study fully analyzes the context and semantics of Chinese comments. With reference to previous studies (Liu and Qi, 2018), this study removes some uncommon emotions contained in the comments and focuses on the two most common discrete emotions in the three branches of the OCC model. The first branch, which deals with the overall conclusion of the event, is the consumer’s self-experience brought about by the “purchase” event. If the perceived result is in line with the consumer’s own mind, the consumer will feel satisfied; otherwise, they will feel disappointed. The consumer’s psychological assessment of the service supplied by the agents, which comprise commodities sellers, logistics, and the e-commerce platform, is the source of the second branch. If consumers feel that the service provided by the agent is better than they expect, they will have the emotion of admiration; otherwise, they will have the emotion of reproach. The third branch comes from the “internal reaction” of consumers as to whether they like the commodity object itself, which includes the appearance, screen, battery, and other attributes of the mobile phone, so as to produce the emotions of love and hate. To categorize the discrete emotions, the simplified OCC model containing the abovementioned six discrete emotions was employed. Figure 1 shows the respective emotions’ recognition pattern diagrams.

### 3.2 Discrete emotion classification

The initial stage in the discrete sentiment categorization of internet comments is data pre-processing. The basic procedure entails removing extraneous data from the annotated corpus and dividing the text into semantically relevant minimum units. First, this research removes illegal characters, converted full-angle characters to half-angle characters, and simplifies traditional Chinese characters. The study then performs a word separation method, which consists of breaking down a string of Chinese characters into distinct words. The study comprehensively compares the existing word segmentation tools, including the word separation tool Jieba, the Chinese lexical analysis system ICTCLAS, and the Chinese lexical analysis toolkit THULAC, and finally selects the Jieba word segmentation tool for word segmentation for its clear code and good scalability. In addition, since there are many colloquial Internet words in Chinese reviews on the Internet, this study utilizes the Internet thesaurus SogouW provided by Sogou Lab to assist with word segmentation to make the word segmentation results more satisfactory. Sogou Lab is the window of external communication for the central research and development team of Sogou Search, and the SogouW Thesaurus is derived from the statistical analysis of Chinese Internet corpus indexed by Sogou Search Engine,^1^ the scale of the Internet corpus involved is more than 100 million pages, with about 150,000 high-frequency words. The final part of the data pre-processing was the removal of stop words. In this study, words contained in the comments that had little to do with sentiment information or thematic information are filtered and removed in order to facilitate sentiment analysis. A total of 2,032 stop words were used in this study, which were finally obtained by collating, integrating, and reprocessing the Baidu stop word list, the Harbin Institute of Technology stop word list, the Sichuan University Machine Intelligence Laboratory stop word list, and other word lists.

In contrast to conventional texts, online product reviews are characterized by variety, complexity, redundancy, and a lack of specification (Xu et al., 2018). Therefore, a central concern of this study is to bring the result of the disambiguation of review texts into a form that can be recognized and processed by computers before a discrete sentiment classification is performed. Commonly used models for text representation include Boolean, probabilistic, vector space, and graph space models. Of these, the vector space model was first proposed by Salton in 1975 (Salton, 1975) and is by far one of the most classical and widely used models of text representation. In this study, the vector space model is adopted to represent the text. In order to avoid the vector space dimension of the text being too large, which would lead to sparse text and a large amount of noise, thus increasing the burden on the subsequent machine learning training process, the study referred to the relevant literature (Yang and Pedersen, 1997; Shang, 2007; Qiu et al., 2008) and compared the most widely utilized feature selection methods, namely, document frequency (DF), mutual information (MI), expected cross entropy (ECE), Chi-square statistics (CHI), information gain (IG), and weight of evidence for text (WET), and finally used the Chi-square statistics to select the features of Chinese comment texts.

According to previous research (Liu and Qi, 2018), this study completes the feature selection process by running cardinality statistics on all sub-datasets corresponding to the six discrete emotions with a positive to negative sample ratio of 1:1, sorting them from largest to smallest based on cardinality values, and removing the top words from the sorted sequence to form a list of feature words corresponding to the six discrete emotions. These word lists are then used to develop a classifier for each discrete emotion through a machine learning approach. To categorize each discrete sentiment implicit in the online reviews, the researchers used Gaussian Bayes (GaussianNB), K-nearest neighbor (KNN), logistic regression (LR), random forest (RF), and support vector machine (SVM).

**Figure 2:**
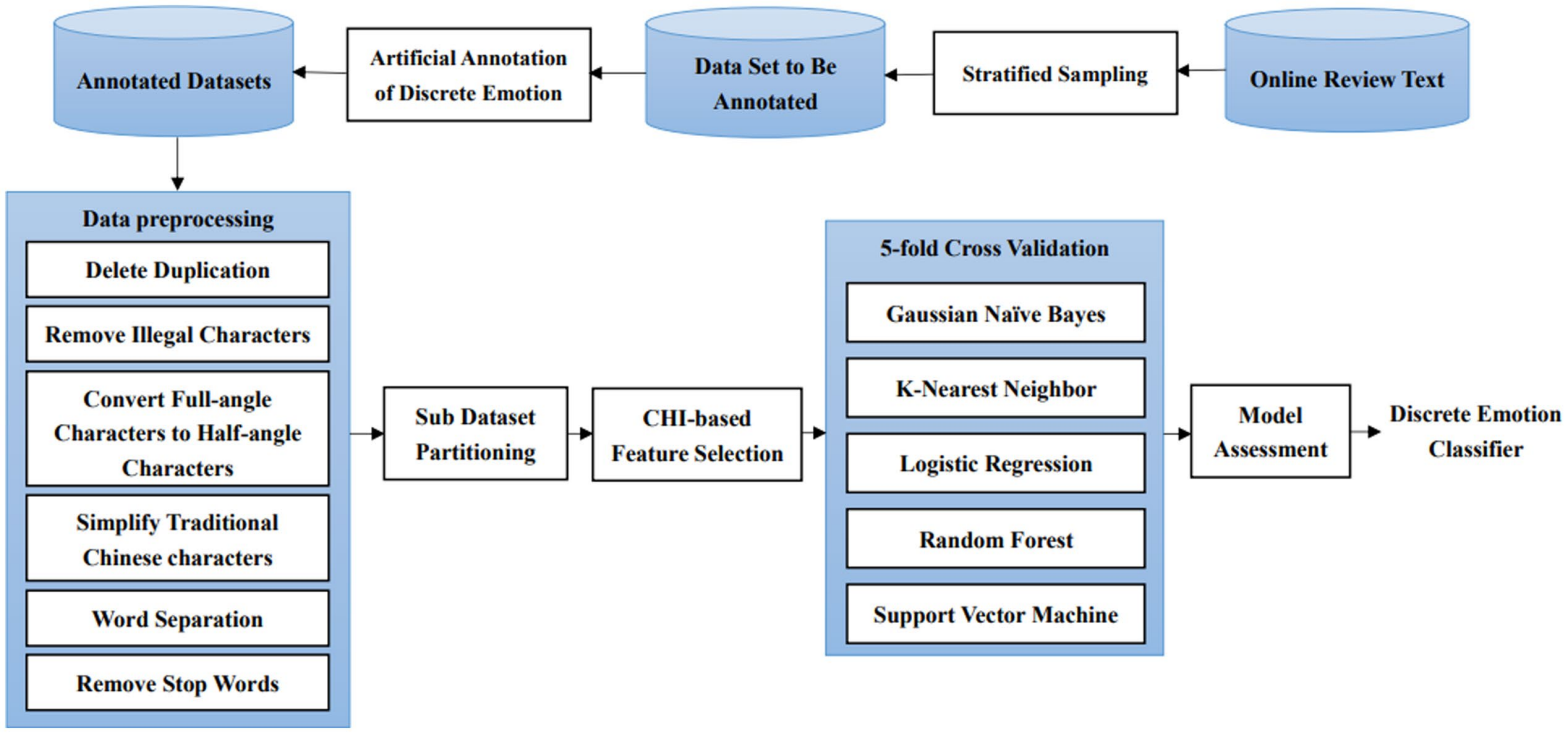
The commentary discrete emotion classification process based on the OCC model.

Due to the large number of sentiment types, a five-fold cross-validation is performed on the six sub-datasets completing the entire annotation and a list of feature words is generated in this study to prevent over-fitting brought about by the complex classification models. That is, each sub-dataset corresponding to a discrete sentiment is divided into five parts, four of which are used as training sample sets and one as a test sample set. According to the above five methods, five cycles of training are conducted, and the average of the five training evaluation metrics is used as the final evaluation result, resulting in a sentiment classifier for the six discrete sentiments of Chinese online reviews (Figure 2).

The most commonly used indicators for evaluating supervised machine learning algorithms are accuracy, recall, precision, and F1 measure. Since F1 measure is the harmonic average of accuracy and recall, it can be regarded as a comprehensive evaluation index of both; the average of the F1 measure at the end of the five training sessions was used as the final evaluation result after the five-fold cross-validation.

Also, as the number of samples corresponding to the six discrete emotions in each sub-dataset varies, the size of the feature word list calculated from the Chi-square statistics also varies. In this study, for each classifier, the first 10, 20, 30, 40, and 50% of Chi-square statistics in different feature word lists are used as feature dimensions, and the peak of the average value of the corresponding F1 measure is used as the F1 measure of the corresponding discrete emotion classifier.

Comparing the experimental results, the LR algorithm-based classifier has good classification results for the six discrete emotions, with an F1 measure of 76% or more, among which the F1 measures of satisfaction, love, and hate emotions even exceed 80%. The worst classification result is achieved by the GaussianNB-based classifier, with an F1 measure of less than 57% for all six discrete emotions, and an F1 measure of only 30.54% for love.

## 4 Dynamic analysis of human behavior on the expression of discrete emotions in online reviews

### 4.1 Statistical analysis of the distribution of reviews with different discrete emotions in different “purchase-comment” time intervals

In order to accurately perform behavioral dynamics analysis of discrete sentiment expressions in online comments, the classifier based on the LR algorithm is shown to be the best at classifying each discrete emotion according to the result of the discrete emotion classification study described above. This study first classifies the remaining unlabeled data of nearly 300,000 reviews by six different discrete sentiment classifiers based on the LR algorithm. The final result is a large corpus of discrete sentiment comments based on the OCC model. Next, this study discusses the group level, which regards the behavior of consumers’ reviews as a whole, without distinguishing consumers and commodities, and analyzes the dynamics of human behavior at different “purchase-comment” time intervals. Firstly, the study captures the time series data of 332,805 Chinese reviews containing the above six discrete emotions, that is, the time when each review publisher purchased the goods ( purchase_time ) and the time at which the review was posted ( review_time ), and gets the “purchase-comment” time interval by calculating the time difference between those two times, that is,


$$x_{i}=review\_time_{i}-purchase\_time_{i}$$

The basic description information of the “purchase-comment” time interval including the six different discrete emotional comments is shown in Table 3.

**Table 3:** Basic data description of “purchase-comment” time interval containing different discrete emotional reviews (measured in days).

Discrete emotion	Number of reviews	Maximum	Minimum	Median	Average	Standard deviation
Satisfaction	178,813	232	0	8	22.74	34.22
Disappointment	38,173	219	0	8	21.73	33.25
Admiration	76,552	214	0	5	17.49	29.98
Reproach	42,773	223	0	6	17.30	28.95
Love	188,999	232	0	7	21.12	33.03
Hate	42,525	223	0	11	26.00	36.11

As can be seen from Table 3, the minimum values of the “purchase-comment” time interval containing six discrete emotions are all zero, which means that consumers receive goods and comment at the same time on the day of purchase. The maximums of the “purchase-comment” time interval containing different emotions are different, but they all exceed 210 days, that is, the consumers comment more than seven months after the purchase of goods. Through comparison, it can also be seen that among the reviews containing admiration and reproach based on the agent, the time intervals between the purchase of goods and the release of reviews are always the shortest, and the median or average value of the time interval is the least among the six discrete emotions, indicating that compared with the purchase event and the goods themselves, the quality of service provided by merchandisers, logistics transport providers, or e-commerce platforms can stimulate consumers to respond faster and give relevant evaluations. At the same time, the “purchase-comment” time of reviews containing the emotion dislike is the longest, with an average of 26 days and a median of 11 days. This shows that when consumers are not satisfied with some attributes of the product itself, consumers generally wait two weeks or even a month to release relevant reviews stating that they do not like the product, which makes these negative reviews on the performance of the product more objective and accurate.

**Figure 3:**
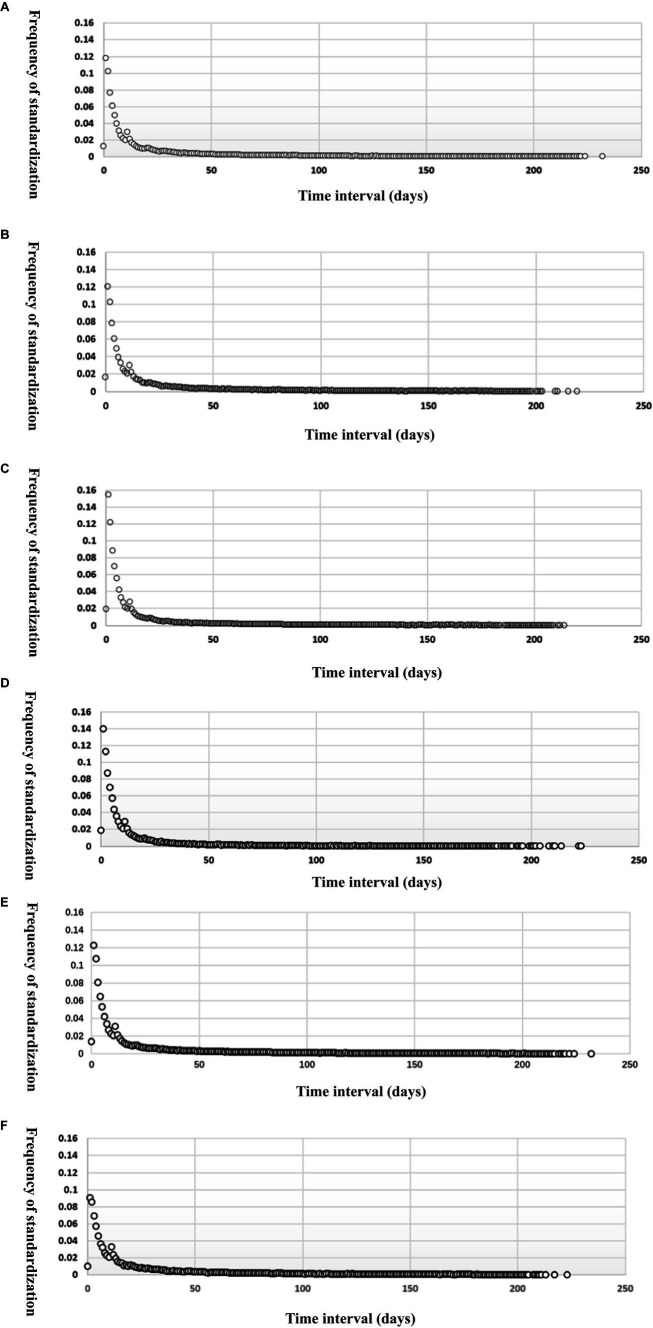
Distribution of reviews containing discrete emotions at different “purchase-comment” time intervals.

From Table 3, we can see the basic descriptive information of the comment data, but the number distribution of reviews in each time interval cannot be displayed. Because the numbers of reviews containing different discrete emotions are different, the study calculates the standardized frequency of review release in different time intervals, and the time interval distribution scatter diagram of different discrete emotional reviews is shown in Figure 3.

**Figure 4:**
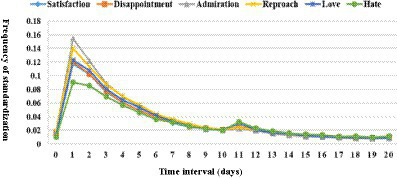
Distribution chart of the release frequency of comments containing discrete emotions at the “purchase-comment” time interval of 0 ~ 20 days.

It can be seen from Figure 3 that no matter what the kind of discrete emotion, the “purchase-comment” time interval distribution graph has a huge tail with obvious “fat-tail” characteristics. However, the standardized frequency of reviews containing different discrete emotions is different within the “purchase-comment” time interval of 0 to 30 days. The shorter the time interval, the greater the difference in the frequency of releasing reviews with different discrete emotions. Zhang (2018), referring to previous research results, divided the “purchase-comment” time intervals into short-term, medium-term, and long-term time intervals, in which the short-term time interval refers to 0 ~ 2 days, the medium-term time interval refers to 3 ~ 19 days, and the long-term time interval refers to 20 ~ 180 days. It was found that the first two types, namely, the number of reviews within 19 days between purchase and comment, accounted for 76.45% of the total number of comments, which is in line with Pareto’s Law. Based on this, this study compares the release frequency of comments containing six different discrete emotions at the “purchase-comment” time interval of 0 ~ 20 days. The comparison results are shown in Figure 4.

Figure 4 shows that within 0 ~ 20 days after the purchase of goods, the first peak of the standardized frequency distribution curve containing different discrete emotional comments appears on the first day after the purchase of goods, in which the comments containing admiration and reproach are published the most frequently, both exceeding 14%, indicating that when the logistics and distribution time of goods is very short, such as receiving goods on the day of purchase or on the second day, the emotion based on the agent can most arouse consumers’ comments, that is, in evaluating the quality of services provided by commodity sellers, logistics transportation, or e-commerce platforms. The descriptive statistics in Table 3 also confirm this conclusion. The third most frequently released reviews contain object-based love. Due to the short time of receiving and using goods, consumers often give a “love” evaluation according to the first impression of goods, such as appearance, color, packaging, and other commodity attributes. Therefore, this evaluation is generally subjective. Among the comments published on the first day after the purchase of goods, the number of comments containing object-based hate is the least, and its standardization frequency is about 9%, indicating that consumers are more cautious in giving negative comments on the goods themselves within a short time after receiving the goods.

In addition, it can be seen from Figure 4 that the six distribution curves corresponding to the six emotions will decrease after reaching the first peak, but the second peak appears on the 11th day after the purchase of goods, especially love and hate based on the objects and satisfaction based on the events. The corresponding curves are more similar to the “bimodal distribution.”

### 4.2 Human behavior dynamics analysis of reviews containing different discrete emotions In different “purchase-comment” time intervals

In order to show the distribution law of time intervals more clearly, this study further analyzes whether this distribution still follows the power-law distribution. In this study, the method proposed by Clauset et al. (2009) fits the power-law distribution through univariate linear regression and ordinary least square methods. The specific analysis process is as follows.

*Step 1*: Assume that the distribution curve of the power-law function is $y=ax^{-b}$ , where a>0 and *y* represents the probability of occurrence of the “purchase-comment” time interval for reviews containing a discrete emotion. It is expressed by the standardized frequency, that is, the ratio of the number of reviews containing the discrete emotion in a “purchase-comment” time interval to the total number of reviews containing the discrete emotion; *x* represents the time interval between consumers’ purchase and comment. The study converts the time interval data in days to hours.

*Step 2*: Take logarithms on both sides of the power-law distribution function $y=ax^{-b}$ at the same time to obtain lny=(−b)lnx+lna . Assuming v=lnx ， u=lny ， A=lna ，the curve equation of power-law distribution function becomes a linear equation u=A+(−b)v .

*Step 3*: Use each set of ordered real number pairs $\left(x_{i},y_{i}\right)$ to find the corresponding $\left(v_{i},u_{i}\right)$ ，where i=1,2,3,…,n , and *n* is the number of “purchase-comment” time intervals containing the above discrete emotional comments.

*Step 4*: Carry out regression by using the least square method to calculate the estimated value $\hat{b}=\frac{\overset{n}{\underset{i=1}{\sum }}v_{i}u_{i}-\left(\overset{n}{\underset{i=1}{\sum }}v_{i}\right)\left(\overset{n}{\underset{i=1}{\sum }}u_{i}\right)}{n\overset{n}{\underset{i=1}{\sum }}v_{i}{\;}^{2}-{\left(\overset{n}{\underset{i=1}{\sum }}v_{i}\right)}^{2}}$ of *b* and the estimated value $\hat{A}=\bar{u}+\hat{b}\bar{v}$ of *A*.

*Step 5*: Get the estimated value $\hat{a}$ of *a* by $\hat{a}=e^{\hat{A}}$ .

**Figure 5:**
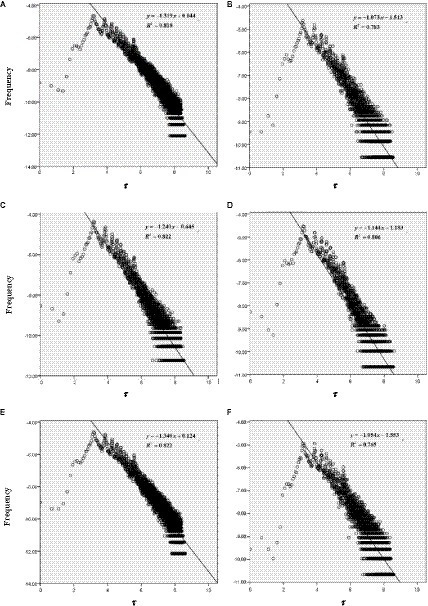
Double logarithmic coordinate scatter fitting diagram of “purchase-comment” time intervals of the reviews containing each discrete emotion.

According to the above five steps, this study performs double logarithm scatter plot processing and linear fitting on the “purchase-comment” time interval data corresponding to six discrete emotions, and the effect is shown in Figure 5.

From Figure 5, it can be seen that excluding the “sagging” head of the curve, the power exponential fit of the time interval scatterplots corresponding to comments containing the six discrete emotions shows a good fit with high values of the decision coefficients $R^{2}$ . Among them, the $R^{2}$ value of the corresponding curves of admiration and love are the largest, both reaching 0.882. The corresponding curve fit for hate has the smallest $R^{2}$ value, but also reaches 0.765. At the same time, it can also be seen that although the power-law indices corresponding to the six discrete emotions are different, their absolute values are concentrated between 1.054 and 1.319, and they are all consistent with the power-law distribution index of human behavior dynamics. The absolute values of the power-law indices corresponding to the six discrete emotions are further ranked in descending order, and the results were love > satisfaction > admiration > reproach > disappointment > hate, that is, the power-law indices corresponding to the three positive discrete emotions are larger than the power-law indices corresponding to the three negative discrete emotions. Figure 5 shows that the first half of the distribution curve of the three positive discrete emotions, especially the curve corresponding to the first 20 days or so, is steeper and the second half is flatter. This means that the percentage of positive reviews made by consumers decreases rapidly with the passage of time in the relative short period of time after the purchase, while the percentage of positive reviews made by consumers on a daily basis declines slowly and changes little. This is also consistent with the reality that consumers are more eager to share their positive emotions after receiving a product, especially their love for the product. However, when consumers have negative emotions about products, services, or the whole purchase event, they may make comments, and because the description of these comment content is relatively specific, commentators need more time to think, carefully organize, and describe them, resulting in a relatively random time for comments. This is why positive discrete emotions have higher power law distributions and steeper drops. From the perspective of three types of emotions based on the event, agent, and object, we can find that the power-law indices of love and hate based on the object have the largest difference, and the former has the largest power-law index among the six discrete emotions while the latter has the smallest power-law index. It means that compared to the whole purchase event or to the services provided by the product seller, logistics and transportation, and the e-commerce platform, the love for the product itself most inspires consumers to express their feelings by posting comments within a short period of time after the purchase, and it decreases quickly within a short period time. The expression of dislike for various aspects of the properties or performance of the commodity itself tends to decrease more slowly in a short period of time and will continue for a relative long period of time. When consumers buy and use the commodity for a long time, they will not tire of expressing their dissatisfaction online when they find defects in the attributes or performance of the commodity.

In fact, in the field of psychology, using the Appraisal Tendency Framework (ATF) theory proposed based on the theory of Emotional Cognitive Evaluation and Motivation Theory, it has been pointed out by many scholars that a specific evaluation dimension or cognitive component is not sufficient for the generation of emotions, and the combination of different cognitive components can promote the generation of emotions (Kuppens et al., 2003), and these cognitive evaluation dimensions related to specific emotions can also have an impact on individual judgment and behavior (Kugler et al., 2012). Some scholars (Tiedens and Linton, 2001; Bagneux et al., 2012) studied the determinacy dimension in cognitive evaluation, pointing out that emotions with high certainty characteristics (such as happiness and anger) can give individuals more certainty, activate human heuristic processing, and influence making high-risk decisions. Roseman (1984) and Lerner and Keltner (2001) conducted studies on the dimensions of responsibility and controllability in cognitive evaluation, pointing out that these two dimensions have a unique role in understanding consumer psychology or behavioral motivation and have a major impact on human decision-making behavior. In this study, among the six discrete emotions based on the OCC model, event-based satisfaction and disappointment belong to low certainty emotions, which are emotions generated by the consumer’s own satisfaction with the entire purchase event, caused by the entire purchase situation and process, and are uncontrollable to the consumer themselves. The admiration and reproach emotions based on intelligent agents belong to high certainty emotions, which are emotions generated by others, such as product sellers, logistics transportation, and e-commerce platforms, whether the services provided are as they wish. Moreover, others are controllable in the generation of event results and should bear responsibility for the generation of event results. Object-based love and hate emotions are both highly deterministic emotions generated by the consumer’s personal love for the product itself and are two emotions that they take responsibility for and can control. Due to the similarity of cognitive elements between certainty and controllability and decision risk assessment, namely, “unknown risk” (judged as unknown risk) and “terrifying risk” (risk beyond personal control), emotions of high certainty, personal control, and self-responsibility can positively reflect purchase risk. This is also why the love emotion can most inspire consumers to express themselves through comments in a short period of time after purchasing the product, and it decreases quickly in a short period of time, and on the contrary, even if consumers have been using the product for a long time after purchasing it, once they discover defects in its performance or attributes, they are likely to express their dislike on the internet without hesitation.

Barabási (2005) and Vázquez et al. (2006) pointed out that when the temporal properties of human behavior obey a power-law distribution, more behaviors or events will occur intensively for a short time and then enter a very long period of silence. This physical statistic describing the degree of frequent activity and prolonged silence over a short period of time is called a paroxysm. Goh and Barabasi (2008) proposed to inscribe the definition of the paroxysmal indicator as shown in the equation:


(1) $$B=\frac{\sigma_{\tau }-m_{\tau }}{\sigma_{\tau }+m_{\tau }}$$

where τ denotes the time interval series of the behavior to be studied, $\sigma_{\tau }$ and $m_{\tau }$ denote the standard deviation and mean of the time interval series τ , respectively, and the value of the paroxysmal indicator *B* is between −1 and 1, with the greater the paroxysm, the more the value of *B* converges to 1. Barabási (2005) and Vázquez et al. (2006) also believed that when a long time interval in human behavioral activity is easily followed by a long time interval, and a short time interval is easily followed by a short time interval, such a time sequence of human behavioral occurrences is usually considered to be memorable. The physical quantity that describes the correlation degree of the adjacent subsequences in the time interval sequence of human behavior is memorability.

The steps of the calculation process are as follows. Firstly, the time series $\left\{t_{1},t_{2},\ldots ,t_{n+1}\right\}$ is formed in chronological order and the two adjacent data in the time series are subtracted to obtain the time interval series $\left\{\tau_{1},\tau_{2},\ldots ,\tau_{n}\right\}$ . The total number is denoted as *n*; the time interval series is then divided into two time interval subseries, $\left\{\tau_{1},\tau_{2},\ldots ,\tau_{n-1}\right\}$ and $\left\{\tau_{2},\tau_{2},\ldots ,\tau_{n}\right\}$ , and the correlation coefficient of these two series can be used to measure the memorability of the series. Goh and Barabasi (2008) proposed the following formula to define the memorability of the time series of behavior occurrence.


(2) $$M=\frac{1}{n_{\tau }-1}\overset{n_{\tau }-1}{\underset{i=1}{\sum }}\frac{\left(\tau_{i}-m_{1}\right)\left(\tau_{i+1}-m_{2}\right)}{\sigma_{1}\sigma_{2}}$$

where $m_{1}$ and $m_{2}$ are the means of the two sequences, $\sigma_{1}$ and $\sigma_{2}$ are the standard deviations of the two sequences, and the value of the memorability index M is between −1 and 1. When M>1 , it means that there is a memory effect, and when M<1 , it means there is an anti-memory effect. The larger the positive value of M , the stronger the memorability.

This study used Equations (1, 2) above to investigate the paroxysms and memorability of the “purchase-comment” time interval sequence of reviews containing different discrete emotions, and the results are shown in Table 4.

**Table 4:** Human behavior dynamics index of “purchase-comment” time interval containing different discrete emotional reviews.

Discrete emotions	Power index	Paroxysmal	Memorability
Satisfaction	−1.319	0.2014	0.0877
Disappointment	−1.073	0.2095	0.0347
Admiration	−1.240	0.2633	0.0642
Reproach	−1.144	0.2521	0.0481
Love	−1.340	0.2199	0.0877
Hate	−1.054	0.1628	0.0273

As can be seen from Table 4, for reviews with different discrete emotions, the “purchase-comment” time intervals are all quite paroxysmal, which may be because some consumers have already formed the habit of timely evaluation during their years of online shopping, so they make comments in a short time after receiving goods, while another number of consumers may be affected by the evaluation reminder function and the evaluation incentive mechanism provided by e-commerce platforms so they also release commodity comments in a short time. In addition, by comparing the paroxysms of “purchase-comment” intervals for reviews containing six discrete emotions, it is found that the values of the paroxysms of admiration and reproach based on the agent are the largest, which are 0.2633 and 0.2521, respectively. The paroxysmal index value of hate based on the object is the smallest, which is 0.128. This result is consistent with the relevant descriptive statistics in Table 3, that is, consumers’ reviews containing admiration and reproach will occur intensively within a short time after purchase, while reviews containing hate will occur in a relatively long time after purchase. The memory index of the time intervals of “purchase-comment” are all close to zero for reviews containing the six different discrete emotions, indicating that each consumer’s purchasing behavior and commenting behavior is an independent individual behavior, and each consumer will comment on the internet according to their own behavioral preferences and habits, according to whether the purchase event brings them pleasure, whether the product seller, logistics and transportation, and services provided by the e-commerce platform bring them comfort, and whether the product itself brings them satisfaction. Consumers do not refer to the previous consumers’ comment time to determine their own comment time, resulting in a weak memory of the time interval between consumers completing commodity purchases and publishing various discrete emotional comments.

## 5 Conclusion

Based on discrete emotion theory, human behavioral dynamics theory, and sentiment analysis methods, the study investigated the temporal characteristics of six different discrete emotions expression, including satisfaction, disappointment, admiration, reproach, love, and hate at the consumer group level. The results show that the distribution of reviews containing the six different discrete emotions in the time intervals of “purchase-comments” follows the power-law distribution and has the time characteristics of human behavior dynamics, that is, “strong paroxysm and weak memory”. However, the power indexes corresponding to different emotions are different, in which the three positive emotions have a higher power index and the three negative emotions have a lower power index, while the power index corresponding to the love emotion is the highest and the power index corresponding to the hate emotion is the lowest. Furthermore, the first peak of the distribution curves for each of the six discrete emotions at different “purchase-comment” time intervals occurs on the first day after purchase and then decreases gradually, but at different rates, with the second peak point of the distribution curves for satisfaction, love, and hate occurring on the 11th day after purchase, and the distribution curves for these three emotions are more similar to the “bimodal distribution.” The study has important theoretical and practical implications, as described below.

### 5.1 Theoretical contribution

This study involved management, computer science, psychology, statistics, human behavior dynamics, and other disciplines, with the characteristics of interdisciplinary and multidisciplinary integration. The comprehensive application of different theories, models, and techniques from different disciplines ensured the in-depth development of the research. Specifically, this study started with the discrete emotion theory of psychology, used the natural language processing technology and machine learning methods in computer science, fully excavated the discrete emotion implied in online Chinese comments, and applied the theories and methods of statistics and human behavior dynamics to the discovery of the temporal characteristics of discrete emotion expression at the level of consumer groups. The research not only expands the existing technology-oriented and behavior-oriented research system in the field of online comment sentiment analysis but also provides a new research direction for User-Generated Content (UGC) behavioral characteristics and knowledge discovery.

Online comment behavior is a typical human online behavior in the e-commerce environment. Unlike the existing analysis of the time interval distribution characteristics of online consumers’ “purchase-comment” based on human behavior dynamics theory, which is mostly based on different types of goods, different types of consumers, etc., this study started from the driving force behind the expression of online comments, that is, emotion, to explore the internal mechanisms of discrete emotion expression in online Chinese comments and analyze the time distribution law of different discrete emotion expressions. From the perspective of different discrete emotional expressions in comments, the analysis of the characteristics of the time interval between purchase and evaluation, which are two typical online behaviors of consumers in the e-commerce environment, is also a new supplement to the study of human online behavior dynamics.

From the perspective of group behavior, this study explored the behavioral dynamics of six different discrete emotions implied in mobile product reviews, including satisfaction, disappointment, admiration, reproach, love, and hate. The results show that the distribution of reviews containing the six discrete emotions at different “purchase-comment” time intervals follows a power-law distribution and has the temporal characteristics of human behavioral dynamics, that is, “strong paroxysms and weak memory,” which affirm the heterogeneity of human behaviors again and indicate that on e-commerce platforms, each consumer’s purchase behavior and comment behavior are independent individual behaviors. Each consumer will express his or her emotional state according to his or her own preferences and habits, in terms of whether the purchase event brings him or her pleasure, whether the service provided by the commodity seller, logistics and transportation, and e-commerce platform brings him or her comfort, and whether the commodity itself brings him or her satisfaction. Each reviewer will not refer to the previous comment time to determine their own comment time, nor will they refer to the previous comment emotions to express their emotional state. An in-depth empirical analysis of the temporal characteristics of emotional expression behavior in consumer reviews can open up a new avenue for research on fake review detection and word-of-mouth communication in online reviews.

### 5.2 Managerial implications

The findings of this study have important implications for understanding consumer sentiment characteristics and further exploring their impact on potential consumers’ purchasing decisions, developers’ updating of products, the development of marketing strategies by marketing teams, and the improvement of services by merchandisers, logistics companies, and e-commerce platforms. On the one hand, reviews containing different discrete emotions play a different but important role for different audiences. For the development and design department of an e-commerce platform, the delicate and positive discrete emotional expressions based on commodities, sellers, logistics and transportation, e-commerce platform, and the whole purchase process in the higher star reviews are significantly presented on the front page of the review, which can promote resonance with potential users and increase the sales of commodities. By studying reviews that contain love emotions, product developers can understand which functions and attributes of commodities are accepted by the majority of consumers; by studying reviews that contain hate emotions, they can improve the product design and increase the functional attributes of the product in the process of modifying the product according to the negative feedback given by users, thus enhancing the competitiveness of the product in the market. As the results of this study indicate that more than anything else, users are most concerned with the acceptability of various attributes of the product itself; in-depth analysis of reviews that contain hate-related emotions is essential to increase brand loyalty and improve overall user satisfaction with the entire purchase process. For merchandising and sales departments, the emotive, persuasive love-related keywords contained in the higher-starred reviews can be used to create advertising and other promotional materials for effective merchandising campaigns, while merchandize sellers, logistics, or e-commerce platforms can use reviews containing reproach-related emotions to improve their services, change their thinking, improve their management, and lay the foundations for renewed user satisfaction.

After analyzing consumer comments over a long period of time (0-240 days) following their purchase, it can be found that the distribution of six discrete emotions in different “purchase-comment” time intervals follows a power law distribution and exhibits the temporal characteristics of human behavioral dynamics - “strong paroxysm and weak memory”. However, a closer look reveals that the expression of the different discrete emotions in the reviews has a different relationship with the time interval between the purchase of the product and the release of the review. Among the comments posted within 0–2 days (short-term time interval) of product purchase, the agent-based reviews containing admission and reproach are the most densely posted, indicating that the services provided by sellers, logistics, and e-commerce platforms have stimulated more consumers to make faster responses and detailed comments. These reviews, especially those containing reproach emotions, should be given more attention by sellers, logistics service providers, and e-commerce platforms, to improve their respective service levels. The reviews made by consumers only after 3–19 days (mid-term time interval) of product purchase are more objective, providing strong assurance for potential consumers to make the right purchase decisions, in addition, due to the clear “bimodal” distribution of Event based satisfaction emotion, Object based love emotion and hate emotion, these reviews provide a more objective and accurate description of the features and functions of the product than the first impression when receiving the product for the first time, and product developers should pay more attention to these reviews to continuously improve product quality and performance. Sellers and product developers should pay more attention to product reviews that are only published after 20 days of purchase (long-term time interval). This is because after purchasing and using a product for a long period of time, consumers who still express dissatisfaction online when they discover defects in certain attributes or performance of the product often have higher expectations for the product and the merchant. Sellers need to do a timely public relations response and crisis management to regain consumer trust, and product development personnel need to conduct detailed analysis and research on related products to effectively solve product quality problems.

### 5.3 Limitations and future research

This study is not without limitations. Using only one product category (mobile phones) from a single platform limits the universality of our results. What discrete emotions are implied in the online reviews of other search-based products or experience-based products and what kind of time distribution characteristics these discrete emotions have deserve the same in-depth investigation. Furthermore, the findings of this study rely on the selection of machine learning methods. More advanced natural language processing technology and more accurate sentiment analysis methods will undoubtedly achieve more accurate classification of discrete emotions in online reviews, more accurately understand the expression of discrete emotional distribution in online user reviews, and thus have a positive impact on potential consumers’ purchase decisions, product developers’ product transformation, marketing teams’ sales strategy development, and service improvement of product sellers, logistics companies, and e-commerce platforms. In future research, in-depth studies on related issues can be conducted.

## Data availability statement

The original contributions presented in the study are included in the article/supplementary material, further inquiries can be directed to the corresponding author.

## Author contributions

LL-n: Conceptualization, Investigation, Writing – original draft, Writing – review & editing, Methodology. QJ-y: Project administration, Supervision, Writing – review & editing. WS-f: Methodology, Visualization, Writing – review & editing. ZZ-p: Formal analysis, Visualization, Writing – review & editing. QQ-x: Investigation, Supervision, Writing – review & editing.

## Footnotes

^1^ https://www.sogou.com
